# Low-cost reversible tandem lens mesoscope for brain imaging in rodents

**DOI:** 10.1117/1.NPh.11.1.014306

**Published:** 2024-03-08

**Authors:** Ashly Jose, Pang Ying Cheung, Zahra Laouby, Frédérique Vanholsbeeck, Juliette E. Cheyne

**Affiliations:** a University of Auckland, Department of Physics, Auckland, New Zealand; b Dodd-Walls Centre for Photonic and Quantum Technologies (DWC), Dunedin, New Zealand; c University of Auckland, Department of Physiology, Auckland, New Zealand

**Keywords:** optics, mesoscope, fluorescence imaging, *in vivo*, calcium imaging, GCaMP

## Abstract

**Significance:**

The development of imaging systems that are cost-efficient and modular is essential for modern neuroscience research.

**Aim:**

In the current study, we designed, developed, and characterized a low-cost reversible tandem lens mesoscope for brain imaging in rodents.

**Approach:**

Using readily available components, we assembled a robust imaging system that is highly efficient and cost-effective. We developed a mesoscope that offers high-resolution structural and functional imaging with cost-effective lenses and CMOS camera.

**Results:**

The reversible tandem lens configuration of the mesoscope offers two fields of view (FOVs), which can be achieved by swapping the objective and imaging lenses. The large FOV configuration of 12.6×10.5  mm provides a spatial resolution up to 4.92  μm, and the small FOV configuration of 6×5  mm provides a resolution of up to 2.46  μm. We demonstrate the efficiency of our system for imaging neuronal calcium activity in both rat and mouse brains *in vivo*.

**Conclusions:**

The careful selection of the mesoscope components ensured its compactness, portability, and versatility, meaning that different types of samples and sample holders can be easily accommodated, enabling a range of different experiments both *in vivo* and *in vitro*. The custom-built reversible FOV mesoscope is cost-effective and was developed for under US$10,000 with excellent performance.

## 1 Introduction

Neuroscience methodologies and fluorescence microscopy are constantly evolving necessitating adaptable imaging solutions. Their development at a low cost requires knowledge of both photonics and neuroscience.

A broad set of imaging techniques is already available to study brain structure and dynamics in rodent models; for example, structural and functional magnetic resonance imaging (MRI) allows non-invasive probing of the brain. For structural imaging, MRI provides high three-dimensional spatial resolution. However, for functional imaging, both the spatial and temporal resolutions of MRI are relatively low, prohibiting the effective imaging of neuronal activity with high precision. Further, these systems have high purchase and maintenance costs that limit extensive usage in preclinical studies.^1^^,^^2^

Widefield fluorescence microscopes are extensively used in laboratories, and a multitude of advancements have improved spatio-temporal resolution, signal-to-noise ratio (SNR), working distance (WD), and field of view (FOV).^3^^,^^4^ A microscope’s FOV is inversely proportional to its total magnification. As a result, combining high resolution and large FOV is a challenge that has been overcome by the development of mesoscopic systems. Enlarging the FOV is associated with aberrations, which can be compensated with the choice of the aperture size of the objective lens. Additionally, the optical resolution depends on the wavelength and F number (≈1/(2NA), where NA is the numerical aperture).^5^ Large FOV widefield microscopes have been utilized for monitoring fluorescence activity from superficial cortical layers of brain.^6^^–^^8^ Concurrently, the increased availability of a wide range of fluorescent probes and LED light sources has made widefield imaging less complex and more versatile.^9^^,^^10^

Several studies demonstrate the effectiveness of custom-built mesoscopes for simultaneous and repeated recording of neuronal calcium activity from distant cortical areas in rodent models.^3^^,^^11^^–^^13^ Primarily, the custom-built tandem-lens epifluorescence mesoscope developed by Ratzlaff and Grinvald^3^ showed potential for *in vivo* imaging by conducting experiments using monkey striate cortex stained with a voltage-sensitive indicator. Additionally, the mesoscope configuration utilizing photography lenses offered several advantages over other existing widefield mesoscopes, such as a larger FOV and higher NA.^3^ Typically, the custom-built designs utilize expensive CMOS cameras with larger or similar sensor dimensions to the FOV to be imaged.^7^^,^^14^ The smaller pixel sizes of these expensive camera systems are responsible for improved digital resolution. Moreover, these requirements for the detection system hinder the development of cost-effective mesoscopes, which offer both large FOV and increased resolution.

Here, we report a newly developed mesoscope adapting the tandem-lens design for high-resolution structural imaging in brain slices and commendable functional imaging *in vivo* that utilizes low-cost lenses and a CMOS camera. This system offers two different FOVs, obtained by interchanging the objective lenses.^15^ We demonstrate the efficiency of our system for imaging neuronal calcium activity in both rat and mouse brains *in vivo*.

## 2 Methods

### 2.1 Imaging Platform

**Fig. 1:**
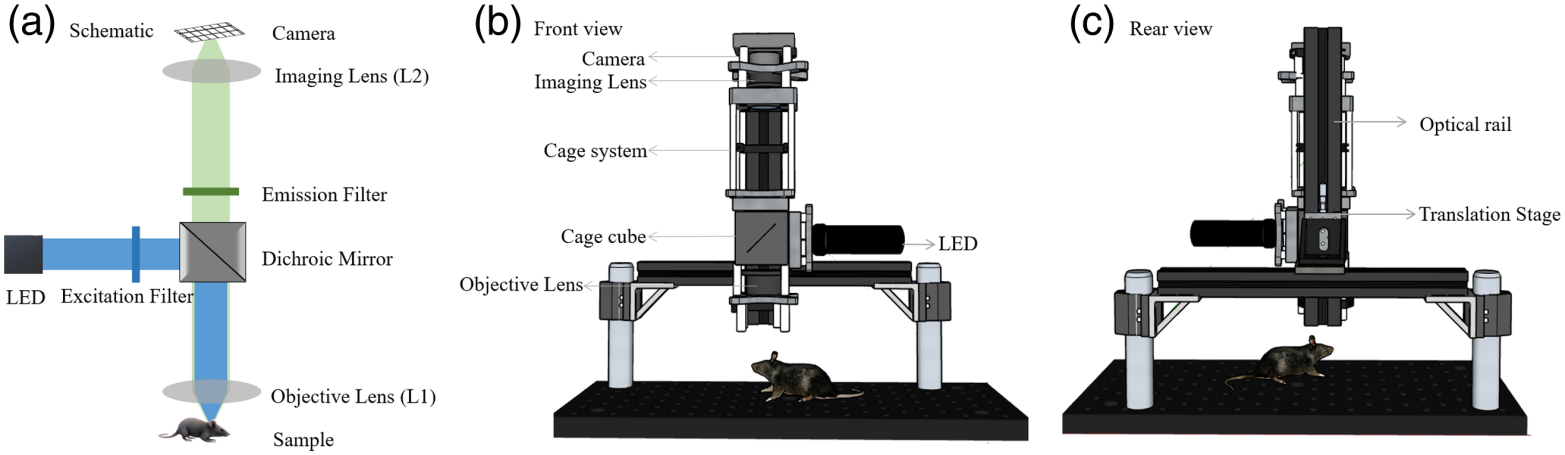
Basic configuration and design of the mesoscope developed using the SketchUp software. (a) The schematic diagram includes the excitation light path in blue containing the LED, the excitation filter, a dichroic mirror, and the objective lens (L1). The emission path, shown in green, encompasses L1, a dichroic mirror, the excitation filter, the imaging lens (L2), and the camera. (b) The camera, lenses, cage cube, and LED in the 60 mm cage system are shown in the front view of the mesoscope. (c) The optical rail and translation stage are shown in the mesoscope’s rear view.

The basic configuration of the mesoscope is illustrated in Fig. 1(a). The key components include an excitation source, a dichroic mirror, optical filters, imaging and objective lenses, and a CMOS camera as detector.

The mesoscope components are assembled using a 60 mm optical cage system for increased flexibility, rigidity, and alignment accuracy. Additional rigidity and freedom of movement for the cage system are provided using a combination of two optical rails (Thorlabs, XT66-200) and dynamically damped mounting posts (Thorlabs, DP14A/M). The optics assembly on the 60 mm cage system is secured on the vertical rail. In addition to the macromovement, achievable through the positioning on the vertical rail, we used a dovetail translation stage (Thorlabs, DTS50/M) that allows a precise z adjustment for 50 mm with 1 mm accuracy. To build a mesoscope with high spatial resolution and large FOVs, we used a compact CMOS camera (Thorlabs, CS505MU) containing a monochrome sensor with an imaging area of 8.4456  mm×7.0656  mm (H×V). We used two fixed-focal-length machine vision camera lenses, L1 (Thorlabs, MVL75M23) and L2 (Thorlabs, MVL50M23), in a tandem-lens configuration with the camera to achieve the desired FOVs. These camera lenses have a design format of 2/3″ to accommodate the sensor format of the CMOS camera.

In addition to the lenses and camera, the optical path of the mesoscope designed for imaging the genetically encoded calcium indicator GCaMP,^16^ consists of an excitation source (Thorlabs, SOLIS 470C), a dichroic mirror (Thorlabs, DMLP490L), and optical filters (Chroma, CT450/70bp and ET520/40m), as illustrated in Fig. 1. The price list of the components used in the system is detailed in Table 3 (Appendix), which demonstrates the cost-effectiveness of the entire system.

**Fig. 2:**
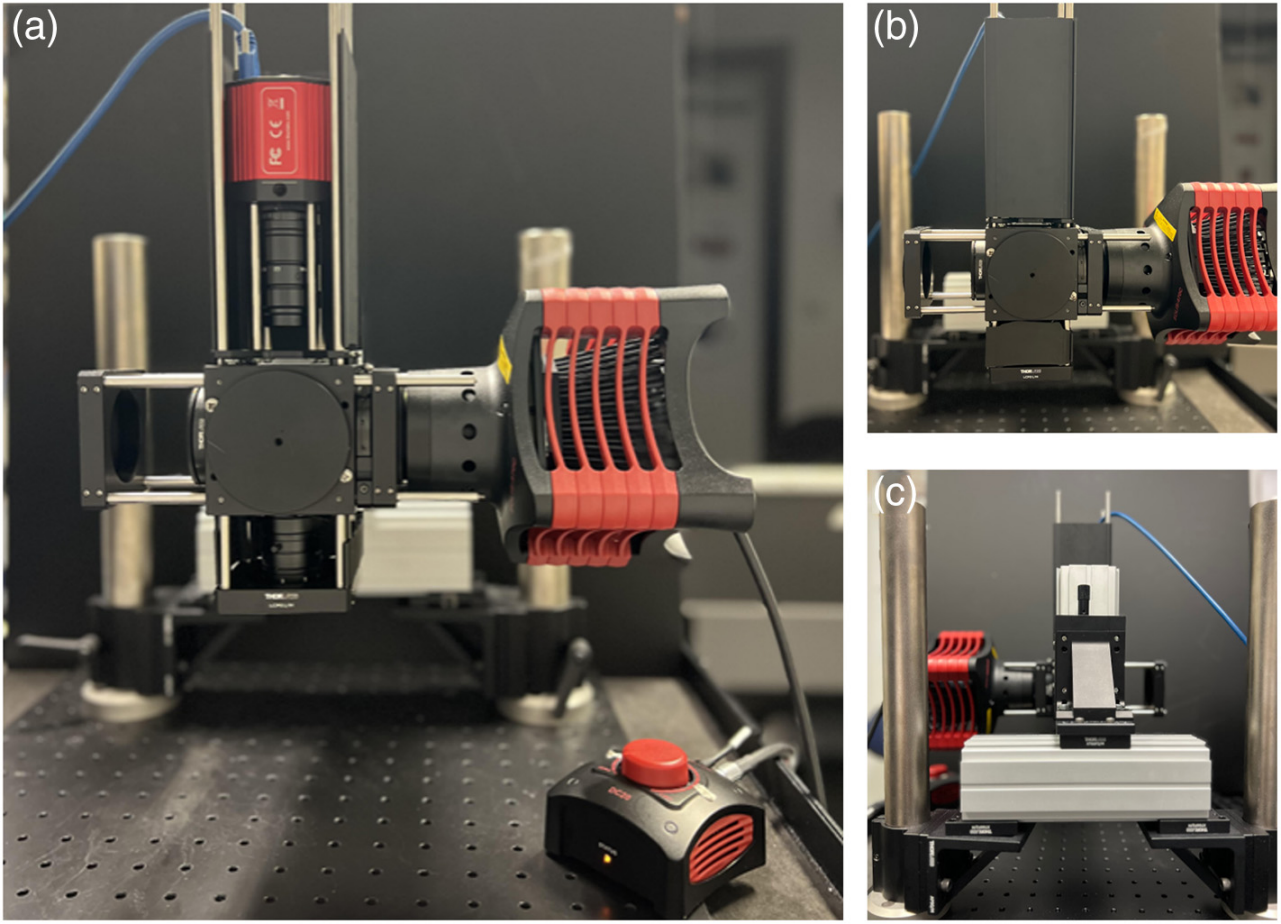
Pictures of the custom built mesoscope. (a) The front view of the cost effective custom built mesoscope without the cage covers displays the arrangement of lenses and camera. (b) The front view of the mesoscope with the cage covers, which isolates the optical path from stray light. (c) The rear view of the mesoscope displaying the arrangement of the optical components such as the posts, rails, and translation stage.

In essence, as demonstrated in Fig. 2, the imaging lens was attached to the camera, and the system of lens and camera was mounted onto the 60 mm cage using a cage mount (Thorlabs, LCP4S). This was attached to the 60 mm cage cube, which contains the dichroic mirror, emission filter, and excitation filter. The objective lens is mounted in the system such that its rear aperture faces the sample. The rear aperture of the imaging lens faces the camera. The LED was mounted onto a 60 mm cage mount (Thorlabs, LCP01/M) and was attached to the cage cube. The objective lens was mounted onto another 60 mm cage mount (Thorlabs, LCP01/M) and was also attached to the cage cube. The cage cube with the cage system was attached to the vertical rail using a clamping platform for rails (Thorlabs, XT66C4). The distance between the imaging lens and the camera is fixed. The distance between the lenses is adjusted to achieve maximum resolution. Increasing the distance between the lenses can result in image clipping and loss of information. The fine adjustment required between the objective lens and the sample is crucial and is performed using the translation stage as mentioned above.

### 2.2 Optical Imaging and Analysis

#### 2.2.1 Transverse sinus injection

All experimental procedures were approved by the University of Auckland Animal Ethics Committee, in accordance with the Animal Welfare Act 1999. Sprague Dawley rats were issued from the Vernon Jansen Unit, University of Auckland. The mice used were the Autism Spectrum Disorder model, Shank3B knockout, wildtype, and heterozygote littermates. This strain of Shank3B mice was established from the purchase from Jackson Laboratory (Stock No: 017688, catalogue: $B6.129\text{-}{\text{Shank}}^{3tm2Gfng}/J$). All animals were housed in a high-containment unit, complying with genetically modified organism containment rules and the Hazardous Substances and New Organisms Act 1996 (HSNO GMD03096 and APP202708).

To drive cortical expression of the genetically encoded calcium indicator GCaMP, we performed transverse sinus injection.^7^^,^^17^ Pups were individually injected on post-natal day 1 (P1). Anesthesia was induced by hypothermia, by placing the pup in an aluminum envelope surrounded by ice for 5 to 10 min. Once anesthesia was confirmed by the lack of movement and reflex response, the pup was transferred to a cold metal plate for the duration of the injection. A small cut was made over the right transverse sinus, and using a stereotaxic frame, a fine glass pipette tip with high resistance was lowered to 400  μm below the skull surface or until blood was seen drawn back into the pipette tip. A total of 4  μL of AAV9-GCaMP7s (pGP-AAV9.Syn.jGCaMP7s.WPRE.SV40, Addgene) was injected into mice and 8  μL to rats slowly using a custom-made injection system.^18^ After the injection, the incision site was closed using a small amount of Vetbond glue (3M, 1469SB Tissue Adhesive). Finally, animals were placed on a heating pad and returned to their home cage once warm and mobile.

#### 2.2.2 Slide imaging

Imaging experiments were conducted to study the performance of the dual FOV mesoscope. The spatial resolution, FOV, and WD of the mesoscope were determined by imaging a standard positive 1951 USAF resolution test target (Thorlabs, R3L3S1P).^14^^,^^19^ The spatial resolution was determined for both configurations using the resolution target positioned at different regions of FOV under the objective lens using the translation stages. The imaging was performed with a frame rate of 10 Hz to record a single frame (n=1) and five frames (n=5).

The fluorescence detection efficiency of the mesoscope was investigated by imaging rat brain slices expressing GCaMP.^20^ Sagittal brain sections were generated from rats that underwent transverse sinus injection as described in Sec. 2.2.1. Briefly, collected brains were post-fixed overnight at 4°C in 4% paraformaldehyde. Fixed brains were then submerged in 20% sucrose and then 30% sucrose. Once the brains sunk, they were dried at room temperature, then instantly frozen using dry ice pellets and stored at −80°C. Frozen brains were sectioned into 50  μm sagittal slices using a microtome at −20°C in optimal cutting temperature cryostat embedding medium.

To enhance visualization of GCaMP, immunolabeling of free-floating rat brain sections was performed. The brain sections were immersed in 1× phosphate-buffered saline (PBS) containing 0.02% azide and stored at 4°C. For GCaMP immunolabeling, brain sections were washed three times with PBS and then immersed for 2 h in a blocking buffer (PBS containing 0.1% triton, 4% normal donkey serum). Sections were incubated in the blocking buffer containing polyclonal rabbit anti-green fluorescence protein (GFP) to label GCaMP positive neurons (1:1000). Sections labeled with anti-GFP were incubated in blocking buffer containing primary anti-GFP overnight at 4°C. Following the incubation with primary antibodies, the sections were washed three times with PBS containing 0.1% triton and incubated for 2 h at room temperature with the secondary antibodies alexa fluor donkey anti-rabbit 488 (1:1000) diluted in the blocking buffer. Sections were washed with PBS and mounted on glass slides. Brain sections were allowed to dry at room temperature before applying the anti-fade (Citifluor) and coverslips. For imaging the slides, a frame rate of 10 Hz was used, and the excitation light at the imaging plane was 4 mW.

#### 2.2.3 In vivo brain imaging

In addition to imaging brain slides, the mesoscope was utilized for conducting *in vivo* imaging experiments in rodent brain. *In vivo* imaging was performed using the large FOV configuration (CF1) and small FOV configuration (CF2) of the mesoscope. Neonatal rats at P9–14 (n=10) and neonatal mice at P8–10 (n=6) were used for these tests. Figure 2 shows the mesoscope used for *in vivo* calcium imaging in animals that were injected with GCaMP at P1. The surface of the skull was exposed by removing the skin and fascia layers above the skull. In addition, the skull transparency was maintained by applying thin layers of cyanoacrylate glue topically, enabling imaging through the skull. For all the experiments, the animals were head-fixed onto a custom-made platform positioned under the objective lens of the mesoscope. The platform was connected to the isoflurane delivery system to induce anesthesia. During *in vivo* imaging, the animals were kept under anesthesia supplied through a nose cone, which delivers 1.5% to 2.5% isoflurane at a flow rate of 1  L/min. Furthermore, the animal’s body temperature was maintained at 37°C throughout the experiments using a feedback-controlled heating pad (Temperature Controller TC-1000, CWE Inc., United States).^21^

The focal plane adjustments of the mesoscope in the z-direction were made using the dovetail translation stage (∼50  mm). The intensity of the excitation light was adjusted using the LED driver. ThorCam software for scientific and compact USB cameras was used for system control and image acquisition. Images were acquired at a frame rate of 10 Hz for visualizing calcium activity. Pixel binning (4×4) was used for reducing the noise and improving the light sensitivity. In addition, pixel binning is also beneficial to reduce file sizes for studies with longer recording time.^22^ Multiple 3 min time-lapse recordings were acquired for each animal. For *in vivo* imaging, the excitation light at the imaging plane was 6 mW.

#### 2.2.4 Image processing and analysis

Recordings were processed using ImageJ^23^ and custom-written MATLAB scripts, and MATLAB apps (developed by Dr. Johan Winnubst). For the test target, the smallest resolved group was compared for the single frame and the average projection of five frames. The WD was measured for both configurations for the focal plane, where the maximum spatial resolution was measured in the center and the worst toward the edges. The NA cannot be directly estimated from the F number of the lenses as they are positioned in an inverted manner for both configurations. Hence, NA of the mesoscope was estimated using the equation NA=n sin θ, where n is the refractive index of the immersion media and θ is the half angle of light acceptance. θ=arctan(D/2f), where D is the rear effective aperture diameter of the lenses and f is the measured WD.^24^ All imaging was performed at the WD optimized using the resolution target. The fluorescence detection efficiency of the mesoscope was calculated in terms of SNR using ImageJ.

For time-lapse *in vivo* recordings, motion artifacts were corrected using NoRMCorre.^25^ Next, normalized fluorescence change stacks (DF/F) were generated using the equation DF/F=(F(t)−F0)/F0), where F(t) represents the fluorescent value of a pixel at a given time (t), and F0 as the mean (all frames) or moving average (200 frame window) fluorescent value. For the figures, we isolated 1 min (600 frames) from the acquired 3 min recordings. The figure traces were generated with F0 as the mean of all frames (600 frames, 1 min). The mean fluorescent changes across were evaluated for four regions of interest (ROIs) for both CF1 and CF2. To display the location of calcium activity on the cortex (in both images and videos), the fluorescent changes in the DF/F stack (mean or moving average) were superimposed onto the mean projection of the raw recordings. Figures were generated using OriginPro software.

## 3 Results

### 3.1 Characterization for Spatial Resolution, FOV, and WD

We constructed a reversible tandem lens mesoscope. To determine the FOV, we calculated the resultant magnification of the lens combinations used for the two configurations. The arrangement (CF1) of L1 (focal length = 75 mm) as an objective lens and L2 (focal length = 50 mm) as an imaging lens provided a magnification of 0.67, i.e., the ratio of the focal lengths. L1 is mounted in the system such that its rear aperture faces the sample. The FOV is 12.6  mm×10.5  mm, which is calculated by multiplying the magnification and imaging area of the sensor. We must point out that the FOV is slightly ellipsoidal due to vignetting. Similarly, the reverse configuration of the lenses (CF2) resulted in a magnification of 1.5 and a smaller FOV of 6  mm×5  mm. In this configuration, L2 is mounted in the system such that its rear aperture faces the sample. In this case, the vignetting is not prominent. The lenses are rearranged to achieve a CF1 or a CF2 based on the imaging requirements.

**Fig. 3:**
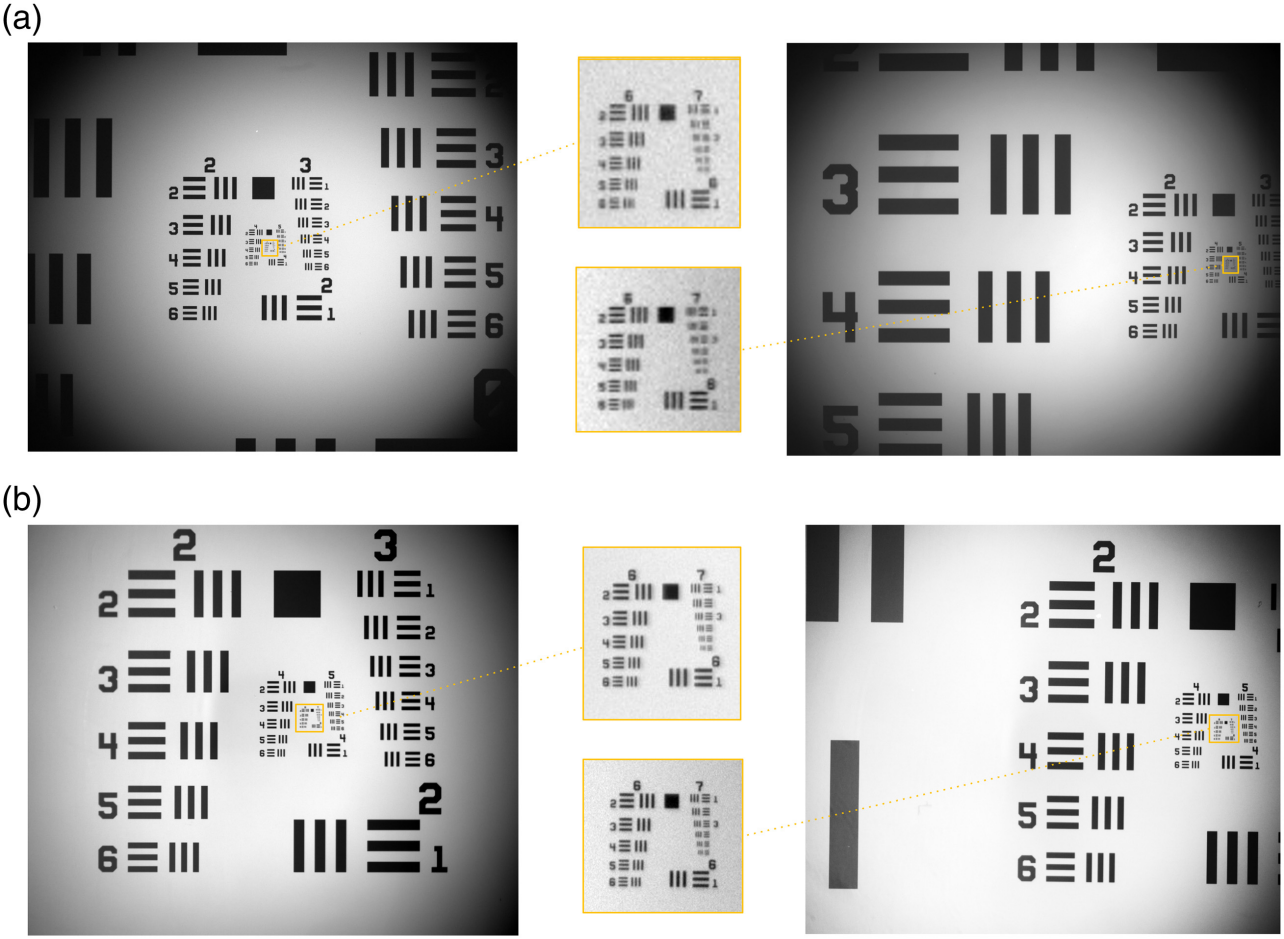
System characterization for measuring the spatial resolution of the mesoscope at different regions of the FOV. The averaged frame (n=5) of the image of the USAF 1951 resolution test target using CF1 (a) and CF2 (b) configuration of the mesoscope. The yellow box indicates the group [6,7] in the respective images at different regions of the FOV, such as in the middle (left) and edge (right) of the FOV.

**Fig. 4:**
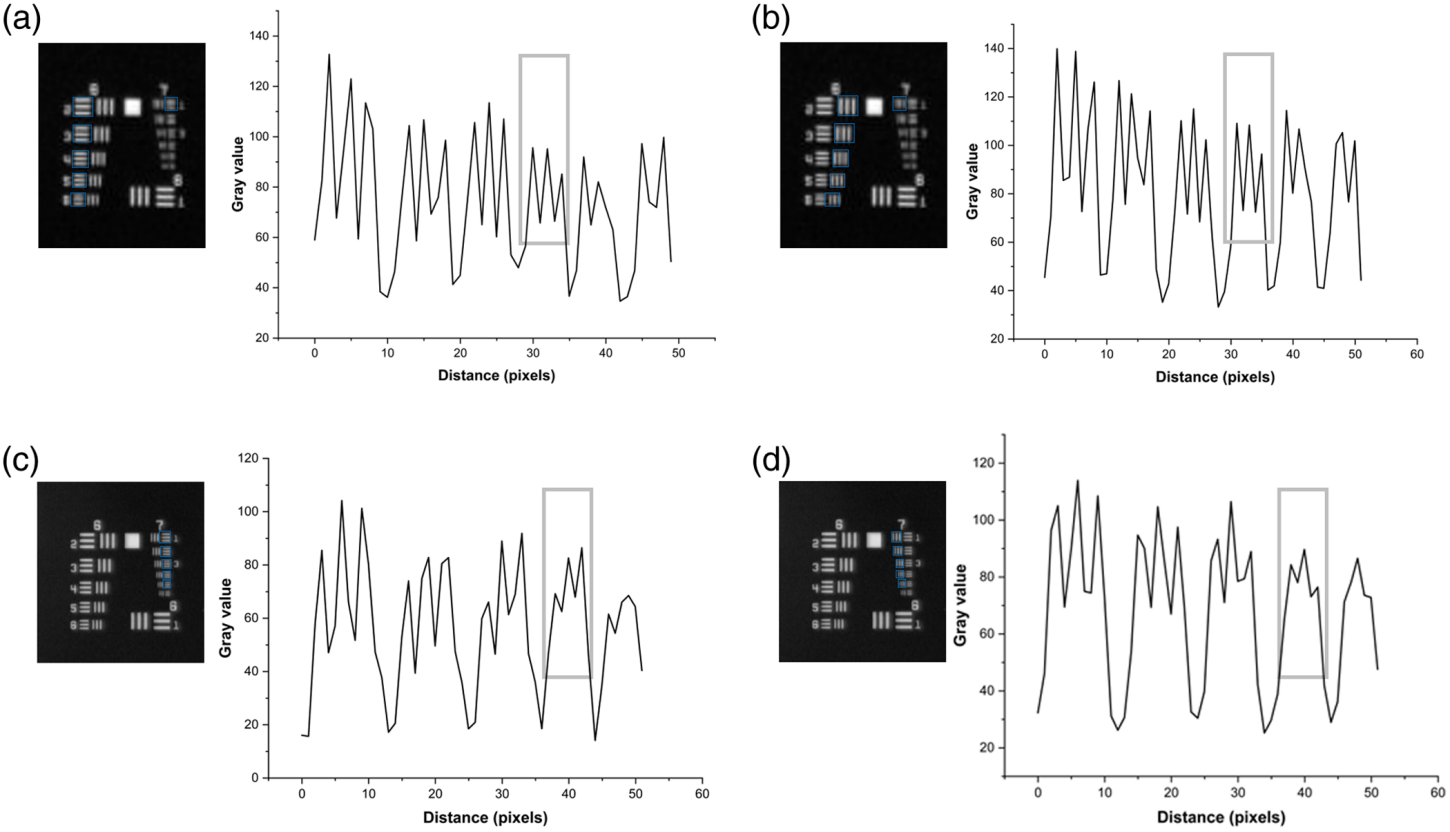
The resolution measurement uses the intensity profile of horizontal and vertical elements of the group [6,7]. The images displaying group [6,7] of the 1951 resolution test target are the invert of averaged frames from Fig. 3 for CF1 and CF2. The intensity profile of horizontal (a) and vertical (b) elements 1 to 6 from group 6 and element 1 from group 7 for CF1. The intensity profile of horizontal (c) and vertical (d) elements 1 to 6 from group 7 for CF2. The rectangle on the plots indicates the maximum resolution for CF1 and CF2 without aberration.

To measure the spatial resolution of CF1 and CF2, images of the resolution test target were acquired at different regions of the FOV. The resolution of a single frame was compared with an average projection of five frames, as given in Fig. 3. The averaged images represented improved resolution for both configurations by reducing the camera noise. Therefore, spatial resolution was evaluated using the averaged images of the resolution target as shown in Fig. 4. With the CF1 configuration, the mesoscope can resolve element 5 in group 6, corresponding to 4.92  μm without aberration across 60% of the FOV. Toward the edges of the FOV, the resolution reduces to 7.81  μm, which corresponds to element 1 in group 6. Similarly, for CF2, the mesoscope resolved element 5 in group 7, corresponding to 2.46  μm without aberration across the full FOV. The WD measured for the best resolution was 1.5 cm for CF1 and 1.0 cm for CF2. Additionally, the NA is calculated for both CF1 and CF2 to be equal to 0.38 and 0.58, respectively. The system characteristics for both configurations of the mesoscope are summarized in Table 1.

**Table 1:** System characteristic for both configurations of the custom-built mesoscope. CF1 and CF2 represent the two different FOV configurations of the mesoscope. M is the magnification, WD is the measured working distances of the system, and NA is the numerical aperture.

Mesoscope configuration	M	FOV (mm)	Resolution (μm)	WD (cm)	NA
CF1	0.67	12.6 × 10.5	4.92	1.5	0.38
CF2	1.5	6.0 × 5.0	2.46	1.0	0.58

### 3.2 Application to Fluorescence Imaging of Brain Slides with GCaMP

**Fig. 5:**
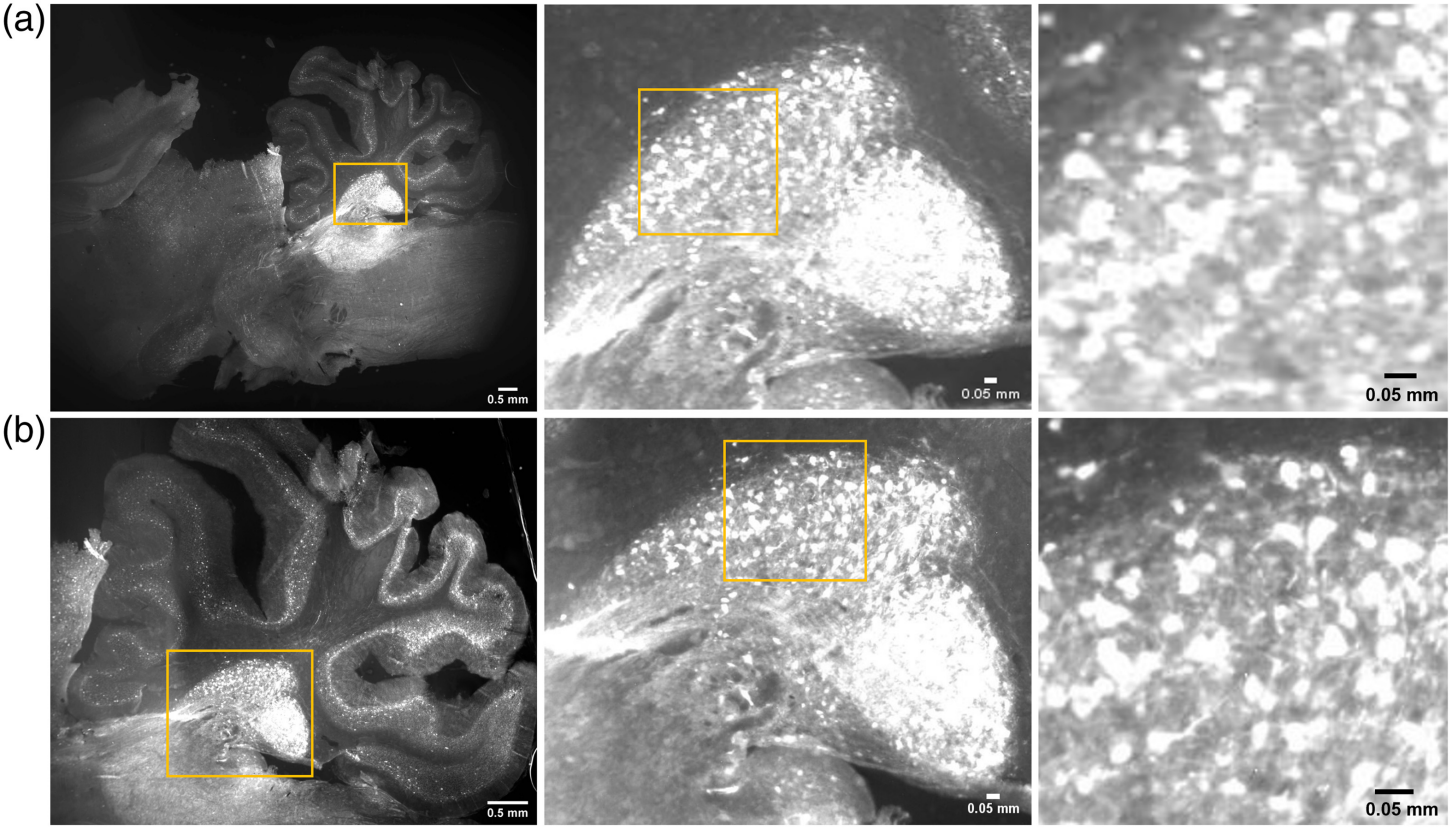
FOV comparison of two configurations (a) CF1 and (b) CF2 of the mesoscope. The zoomed image of the region marked yellow is represented adjacent to the respective images. The fluorescence images were captured using the same brain slide of sagittal sections of a rat brain. The image of the cerebellum imaged using CF2 shows improved SNR under the same illumination.

As a potential application, we used the mesoscope to image brain sections with GCaMP expression under various illumination conditions. The mesoscopic images of the brain slides using CF1 and CF2 are shown in Fig. 5. The images show that a larger region is imaged under CF1 than CF2. However, the cerebellum shows improved SNR under CF2 than CF1 for the same illumination. Also, the SNR calculated for the system was above 10 for the acquired images. The images clearly demonstrated the regions emitting fluorescence in the brain section, showing the efficiency of fluorescence detection of the mesoscope.

### 3.3 Application to In Vivo Fluorescence Imaging of Rodent Brains

**Fig. 6:**
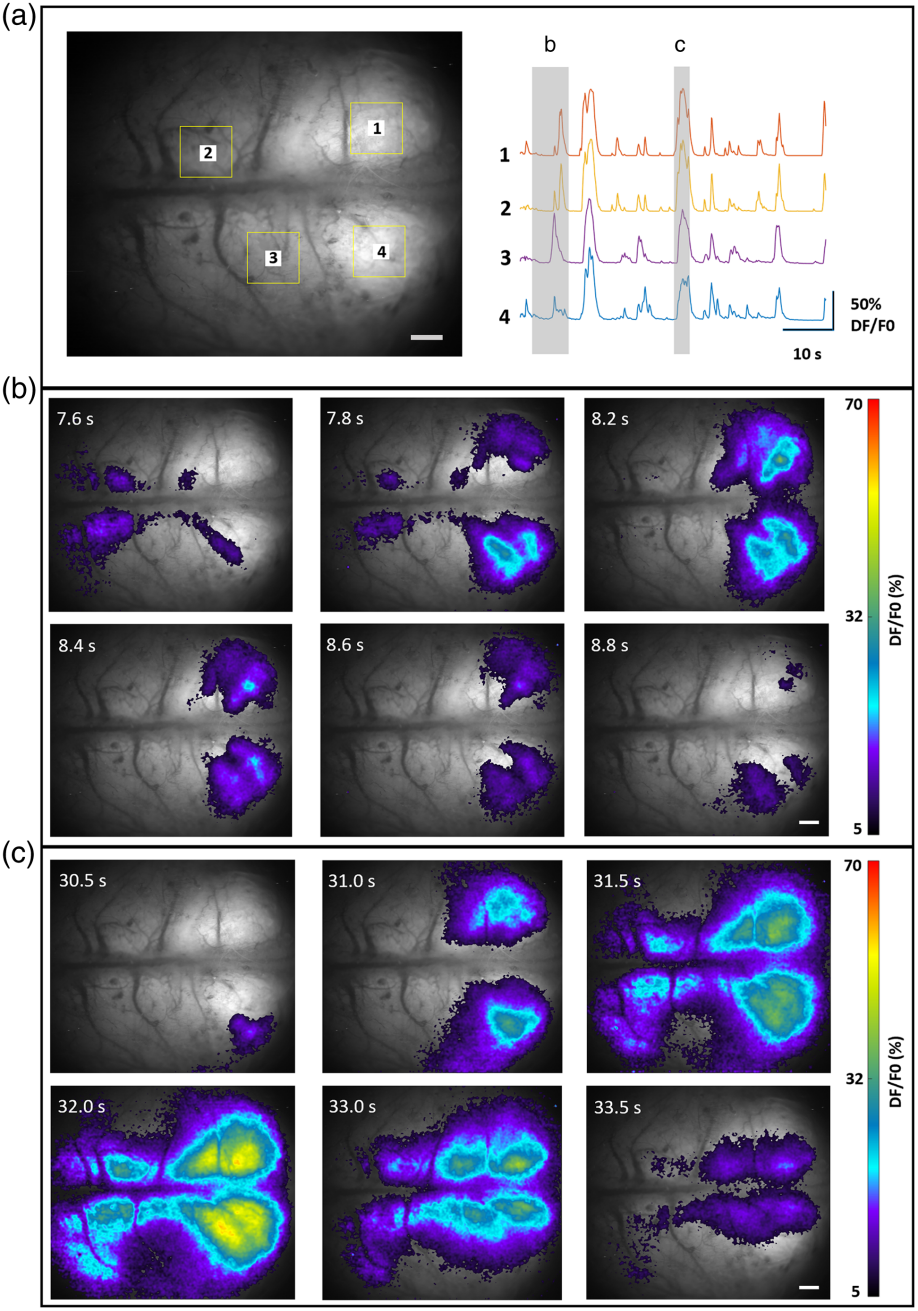
Mesoscopic imaging of brain activity in neonatal rat using CF1. Fluorescence activity from diverse brain regions was recorded using CF1 with FOV 12.6×10.5  mm. (a) Spontaneous calcium activity recorded over 600 frames is represented as fluorescent traces for two different events (ROIs indicated with yellow squares). The strong intensity distribution (b) and lower intensity distribution (c) at various regions in the brain and corresponding time scale are illustrated. The color bar represents calcium intensity profile for minimum signal intensity to maximum signal intensity. The scale bar is 1.0 mm (Video 1, MP4, 2.48 MB [URL: https://doi.org/10.1117/1.NPh.11.1.014306.s1]).

**Fig. 7:**
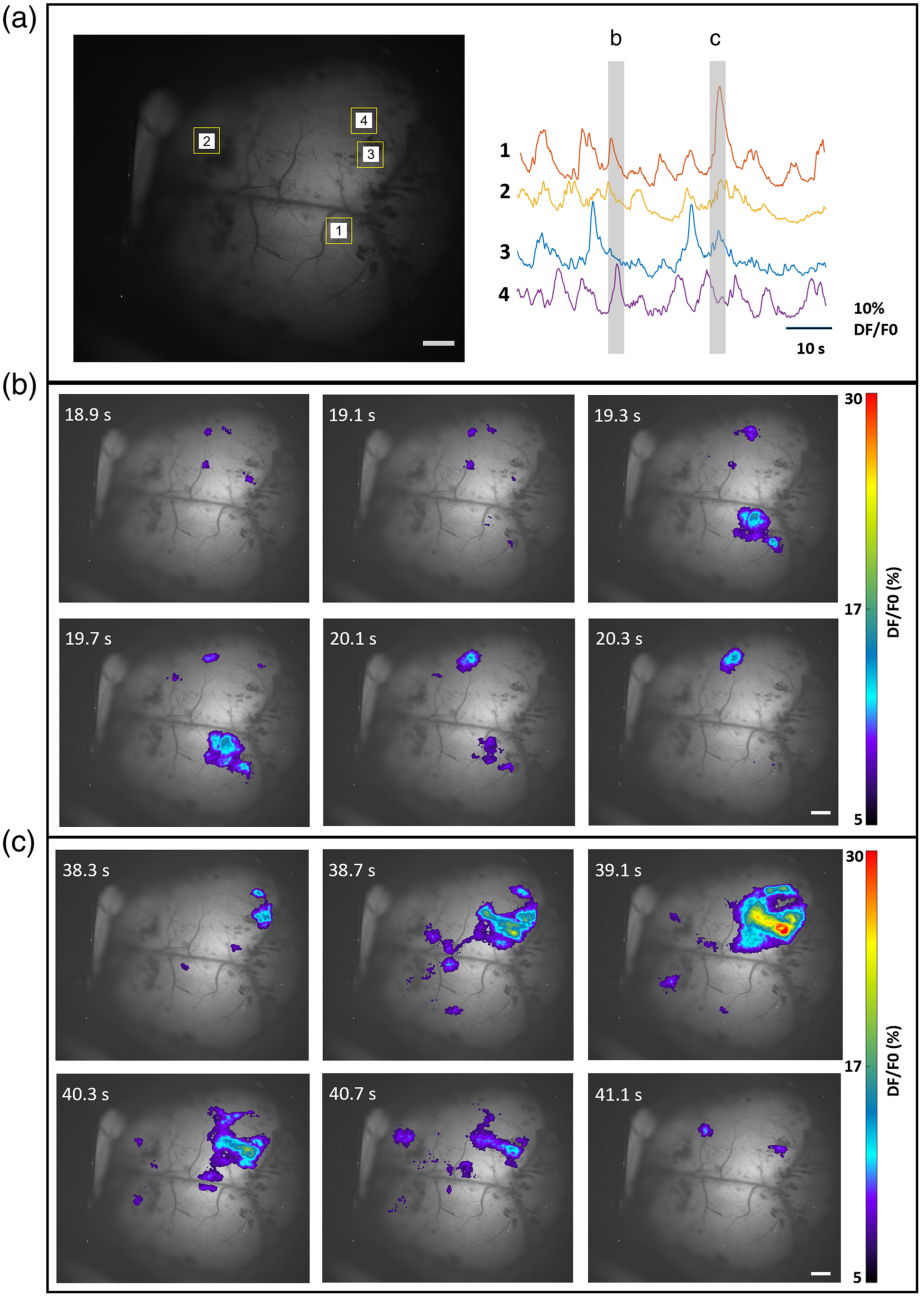
Mesoscopic imaging of brain activity in neonatal mouse using CF1. Fluorescence activity from diverse brain regions was recorded using CF1 with FOV 12.6×10.5  mm. (a) Spontaneous calcium activity recorded over 600 frames is represented as fluorescent traces for two different events (ROIs indicated with yellow squares). The strong intensity distribution (b) and lower intensity distribution (c) at various regions in the brain and corresponding time scales are illustrated. The color bar represents calcium intensity profile for minimum signal intensity to maximum signal intensity. The scale bar is 1.0 mm (Video 2, MP4, 2.15 MB [URL: https://doi.org/10.1117/1.NPh.11.1.014306.s2]).

**Fig. 8:**
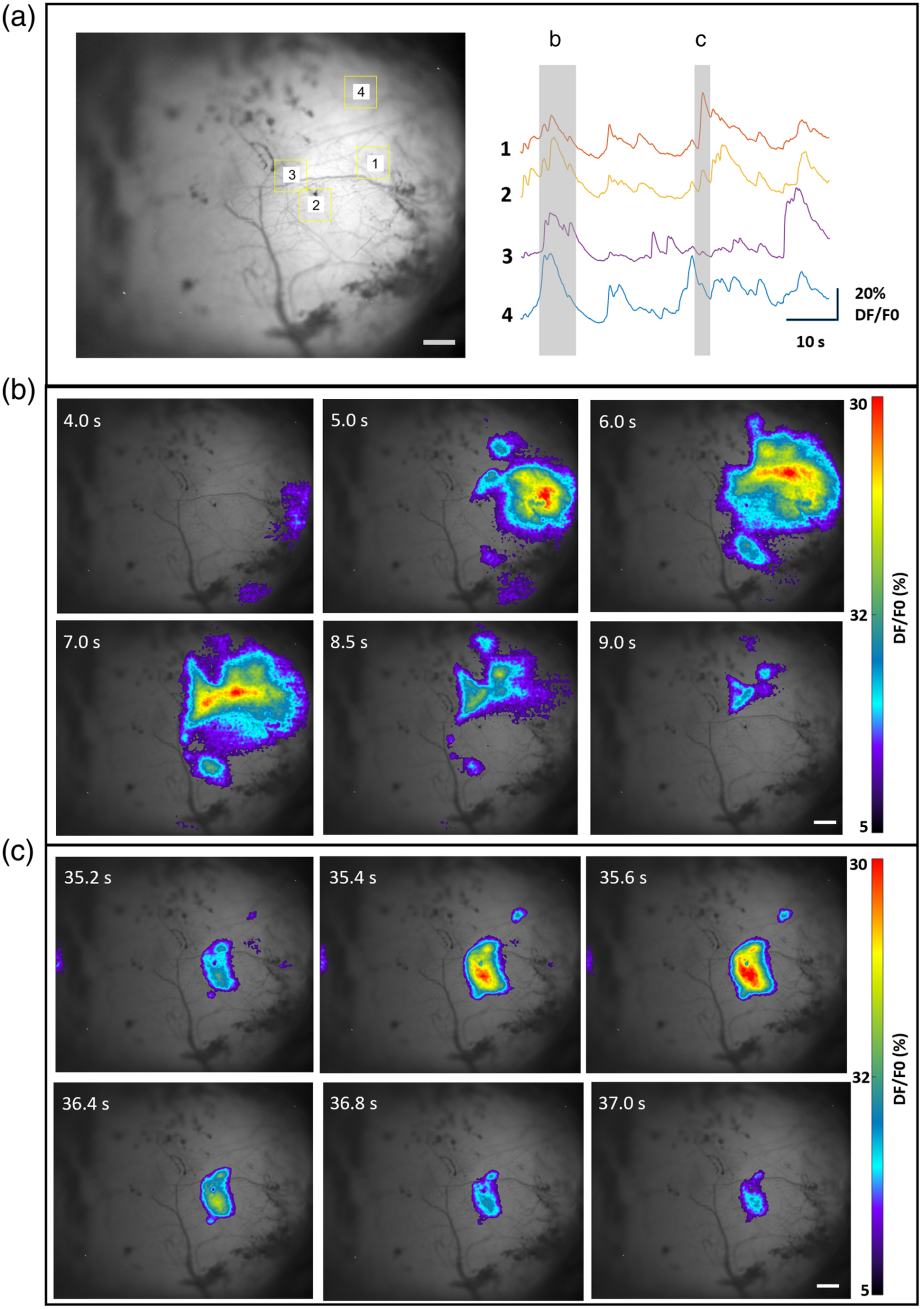
Mesoscopic imaging of brain activity in neonatal mouse using CF2. Fluorescence activity from diverse brain regions was recorded using CF2 with FOV 6.0×5.0  mm. (a) Spontaneous calcium activity recorded over 600 frames is represented as fluorescent traces for two different events (ROIs indicated with yellow squares). The strong intensity distribution (b) and lower intensity distribution (c) at various regions in the brain and corresponding time scale are illustrated. The color bar represents calcium intensity profile for minimum signal intensity to maximum signal intensity. The scale bar is 0.5 mm (Video 3, MP4, 773 KB [URL: https://doi.org/10.1117/1.NPh.11.1.014306.s3]).

The fluorescence images acquired from the brain slides can provide qualitative information on the structures within the resolution limit as described in Sec. 2.2. To detect spontaneous neuronal activity, the mesoscope was utilized to image rodent brains *in vivo*, as shown in Figs. 6–8. Recordings were performed through the skull of rodents using both FOVs of the mesoscope.

Figures 6(a) and 7(a) show an average intensity projection of fluorescence images acquired using CF1. The figure demonstrates the ability of CF1 to record fluorescence from the entire cortical surface of the neonatal rat and mouse brain. Figure 6(a) represents ROIs indicated with a yellow square, showing the spontaneous calcium activity as varying fluorescent signal across 600 frames from rat brain. Similarly, Fig. 7(a) shows calcium activity recorded from a mouse brain cortex from selected ROIs in the FOV (ROI indicated with yellow squares). The spontaneous activity is depicted for two different events for both cases and is represented in Figs. 6(b), 6(c), 7(b), and 7(c). The fluorescence intensity distribution corresponding to calcium activity at various brain regions is depicted as time-varying and spatially heterogeneous signals. The color bar in the figures represents the calcium intensity profile for minimum and maximum signal intensity, respectively. The scale bar is 1.0 mm for both sets of images.

Similarly, in Fig. 8(a), ROIs with varying calcium signaling and corresponding fluorescent traces are depicted. Figures 8(b) and 8(c) show fluorescence generated from different parts of the selected brain region of the neonatal mouse. The FOV of CF2 enabled capturing the fluorescence emission from a mouse brain region with dimension 6×5  mm.

## 4 Discussion

Here, we report the custom development of a mesoscope using a reverse tandem-lens arrangement of machine vision lenses in combination with a low-cost CMOS camera. The camera offers high temporal and spatial resolution, in addition to high sensitivity for wavelengths between 525 and 580 nm. Our mesoscope offers two FOVs with an easily reversible configuration of lenses. Reversing the configuration is straightforward and does not require optics alignment expertise. The researcher can change the order of objective and imaging lenses to enable the small FOV during an experiment if they prefer improved resolution over a larger FOV. The assembly of this robust system using off-shelf components available in most optics labs has proved to be highly efficient and cost-effective. The LED source used for exciting the specific fluorophore in the study can also be replaced easily depending on the central wavelength of excitation required for exciting the fluorophore. In addition to switching the excitation source, the excitation and emission filters tailored for the source and fluorophore should also be used in the system. The careful selection of the mesoscope components ensured its compactness, portability, and versatility, meaning that different types of samples and sample holders can be easily accommodated in a range of environments. Although the current design only incorporates a single optical path, the collimated light path between the lenses can accommodate additional optical paths for isolating or studying non-neuronal signals.^3^^,^^15^^,^^26^

Table 2 demonstrates a comparison between system specifications of various mesoscopes. Our mesoscope offers WDs of 1.5 and 1.0 cm for the two FOVs. Longer WDs are more suitable for incorporating electrophysiological and optogenetic measurements simultaneously during fluorescence imaging for analyzing cellular and network activity.^12^^,^^31^^,^^32^ The WD of the mesoscope could be improved by reducing the NA of the system by choosing a different combination of lenses with increased flange focal distances.^11^ The reduced NA can result in reduced light gathering and decreased resolution, hence decreased SNR and contrast. The closed optical path of the mesoscope is suitable for experiments involving visual stimulation. Additional noise isolation and prevention of stray light entering the objective can be achieved using a light-shielding cone tailored for the WD of the mesoscope configuration.^11^

**Table 2:** Comparison between system specifications of various mesoscopes.

Microscope	Maximum FOV (mm)	Resolution (μm)	Camera type	Sensor size of camera (mm)	Quantum efficiency of camera	Frame rate (Hz)	WD (cm)	NA
Our mesoscope	CF1: 12.6 × 10.5	4.92	Kiralux 5.0 MP	8.45 × 7.07	72% at 525 to 580 nm	35	1.5	0.38
							1.0	0.58
	CF2: 6 × 5	2.46	CMOS Thorlabs					
MacDowell and Buschman^27^	30 × 20	34 per pixel	Optimos CMOS	12.48 × 7.02	55% at 600 nm	13.3	NA	NA
			Photometrics					
Cramer et al.^28^	12 × 12	18.5 per pixel	Interline CCD	7.32 × 7.32	NA	25	4.4	NA
			Adimec					
Musall et al.^29^	12.5 × 10.5	20 per pixel	Pco.edge 5.5 s CMOS PCO	16.64 × 14.04	60%	30	NA	NA
Barson et al.^7^	13.3 × 13.3	6.5 pixel size	Pco.edge 4.2 s CMOS	13.3 × 13.3	82%	15	5.6	0.25
			PCO					
Xiao et al.^22^	9 × 9	68 per pixel	M60 Pantera CCD	12.28 × 12.28	NA	10 to 50	NA	NA
			Dalsa					
Murphy et al.^30^	10.5 -10.75 × 10.5 -10.75	3.45 pixel size	RPi Camera	5.02 × 3.75	NA	30	1.5	NA
			Wave Share Electronics					
Couto et al.^11^	17.1 × 14.4	20 per pixel	Pco.edge 5.5 s CMOS	16.64 × 14.04	60%	30	>4.0	0.35
			PCO					

The system demonstrated high spatial resolution in images of a resolution test target and brain slides. In addition, the preliminary *in vivo* imaging test results demonstrated high temporal resolution for accurately capturing various spatial events from the cortex region of rat and mouse models. The resolution of measurement of CF1 reports the best and worst resolution, whereas the spatial resolution remains the same for CF2 at several points across the FOV. The dominance of vignetting and the resultant reduction in resolution at the periphery of the mesoscope’s FOV requires compensation. The compensation involves using corrected eyepieces, optimizing Kohler illumination, reducing the aperture size by trading off the FOV, and using image processing methods.^33^^–^^36^ Additionally, the spatial resolution measurement using a resolution test target indicates the presence of astigmatism in CF1. Astigmatism is not visible in the CF2. Astigmatism in the images could be due to the lack of precision in the orientation of the optics in the system, such as the dichroic mirror and due to the dimension of the camera sensor.

CF2 also demonstrated increased fluorescence efficiency in imaging brain slides. Although CF2 offered single-cell resolution for imaging brain slides, the *in vivo* imaging performed through a cranial window opening in the skull did not result in cellular resolution, even for imaging performed without pixel binning. The lack of cellular resolution using CF2 is due to several factors such as the increased depth of the imaged neurons, increased light scattering, and/or widespread but low expression of our calcium sensor. It may be possible to achieve cellular resolution *in vivo* if these effects can be compensated. Additionally, the inverse proportionality of the mesoscope’s FOV to its total magnification also plays a crucial role. CF2 with higher magnification offers lower FOV and *vice versa* for CF1. Although a camera sensor dimension larger than FOV is favorable for providing improved resolution across the FOV, for *in vivo* imaging, the edges of the FOV are not usually analyzed due to the shape and curvature of the brain.

The cost of the system can be further reduced by switching to cost-effective filters from Thorlabs, such as FGB7S (excitation path) and FGV9S (emission path). However, the percentage of transmission of the respective wavelength being too low and the reduced filtering of excitation light from the emission light resulted in low-quality images. Furthermore, the cost of the mesoscope can also be reduced by opting for a cheaper Raspberry Pi camera. Although these designs proved to be useful for functional imaging in mice, these could result in low-resolution images, specifically for *in vitro* imaging due to the low sensitivity and reduced noise filtering of such cameras.^30^ Additionally, alternative solutions for excitation sources such as M470L5-C1 from Thorlabs can also be considered for similar applications to reduce the overall developmental cost of the mesoscope.

## 5 Conclusions

The custom-built mesoscope offers two FOVs with an easily reversible configuration. The assembly of this robust system using off-shelf components available in most optics labs has proved to be highly efficient and cost-effective. The mesoscope offers a high sensitivity for its large FOV, comparable with similar systems with reverse tandem-lens configuration used for cortex-wide imaging. To the best of our knowledge, the system characteristics of the mesoscope, developed under US$10,000, including the detection system, offer superior performance compared to similar custom-built large FOV widefield mesoscopes.

## 6 Appendix: Component Cost

Components involved in the design of the mesoscope are listed in Table 3.

**Table 3:** List of components used in the construction of the mesoscope and their cost as per the year 2021.

Part no.	Part, number of parts used (company)	Cost (USD)
ER4-P4	Cage assembly rods 4″ long, 4 pack (1) (Thorlabs)	$27.43
ER1.5-P4	Cage assembly rods 1.5″ long, 4 pack (1) (Thorlabs)	$22.59
ER3-P4	Cage assembly rods 3″ long, 4 pack (1) (Thorlabs)	$25.5
LC6W	60 mm cage cube (1) (Thorlabs)	$154.08
LB3C/M	Mounting platform for 60 mm cage cube (1) (Thorlabs)	$51.78
DMLP490L	Longpass dichroic mirror 490 nm cut-on (1) (Thorlabs)	$349.24
LB5C1	Optic mount for 60 mm cage cube (1) (Thorlabs)	$64.20
AP90/M	Right-angle mounting plates (3) (Thorlabs)	$400.5
LCP4S	30 to 60 mm cage plate adapter 4 mm thick (1) (Thorlabs)	$42.19
LCP01/M	60 mm cage plate (2) (Thorlabs)	$86.74
SM1A10	Adapter with external SM1 threads (2) (Thorlabs)	$42.76
XT66-200	66 mm construction rail L=200 mm (2) (Thorlabs)	$118.14
XT66P2/M	Rail carriage for 66 mm rails with M4 & M6 taps (4) (Thorlabs)	$312.72
XT66C4	Clamping platform for 66 mm rails (1) (Thorlabs)	$32.61
C60L24	60 mm cage system cover 24″ long, pack of 4 (1) (Thorlabs)	$31.64
CS505MU	Kiralux 5.0 MP color CMOS camera (1) (Thorlabs)	$2,658.95
SOLIS470C	High-power LED for microscopy 470 nm (blue) (1) (Thorlabs)	$1,406.85
DC20	High-power driver for solis LEDs (1) (Thorlabs)	$553.89
MVL50M23	50 mm lens EFL f/2.8 (1) (Thorlabs)	$223.70
MVL75M23	75 mm lens EFL f/2.5 (1) (Thorlabs)	$227.12
DTS50/M	50 mm dovetail translation stage M6 × 1.0 Taps (1) (Thorlabs)	$232.83
DP14A/M	Dynamically damped post 14″ long metric (2) (Thorlabs)	$509.72
C1511/M	Post mounting clamp 63.5 mm × 63.5 mm metric (2) (Thorlabs)	$155.50
C15QR/M	Quick release handle 51.4 mm long (2) (Thorlabs)	$22.14
CT450/70bp	50 mm dia mounted Filter (1) (Chroma)	$825.00
ET520/40m	50 mm dia mounted Filter (1) (Chroma)	$825.00
	Total cost	$9402.82

## Supplementary Material







## Data Availability

Data are available on Figshare (https://doi.org/10.17608/k6.auckland.c.6574579). Code is available on GitHub (https://github.com/juliettecheyne/cheyne_lab).
